# What proportion of women presenting to the emergency department with early pregnancy bleeding receive appropriate care?

**DOI:** 10.1111/1742-6723.14507

**Published:** 2024-10-07

**Authors:** Baylie Trostian, Andrea McCloughen, Kate Curtis

**Affiliations:** ^1^ Susan Wakil School of Nursing and Midwifery, Faculty of Medicine and Health University of Sydney Sydney New South Wales Australia; ^2^ Emergency Department Wollongong Hospital Wollongong New South Wales Australia

**Keywords:** appropriate care, bleeding, early pregnancy, emergency department, nursing

## Abstract

**Objective:**

To determine the proportion of women presenting to EDs across a regional health district, with early pregnancy bleeding, who received appropriate care.

**Methods:**

Retrospective cohort review of linked data from five data sets: ED, pathology, radiology, costs and non‐admitted/outpatient. Data collected from five EDs between January 2011 and December 2020, across one health district in NSW, Australia, with 150 000 annual ED presentations. Management received by women of reproductive age, with early pregnancy (<20 weeks gestation) bleeding was compared to seven indicators for recommended care. Indicators included blood tests, psychosocial support, administration of Rhesus D immunoglobulin and US. Indicators were determined by a systematic analysis of published primary research, expert consensus clinical practice guidelines and literature reviews on initial assessment, intervention and diagnostics for women with early pregnancy bleeding.

**Results:**

There was no evidence of almost one third of women (*n* = 3661, 29.4%) receiving any indicators and 54 (0.4%) received five or more indicators of appropriate care. Presentations to rural facility had the lowest number and proportion of indicators being performed (*n* = 603, 58.0% for no indicators). Cost increased with the number of indicators. Over the study period, the proportion of all indicators being performed increased, and indicator six – psychosocial support referral or care had the biggest growth (almost 500%).

**Conclusions:**

Variation in care for women presenting with early pregnancy bleeding to ED was identified. There is an evidence‐practice gap and need for inquiry into barriers and facilitators to prescribed clinical practice for this population.

## Graphical abstract

Vaginal bleeding the most frequent early pregnancy complication, and a common reason for women to present to the ED. Care received can have immediate and long‐term health impacts. Our 10‐year linked data study, across five EDs found that only approximately 10% received appropriate care.

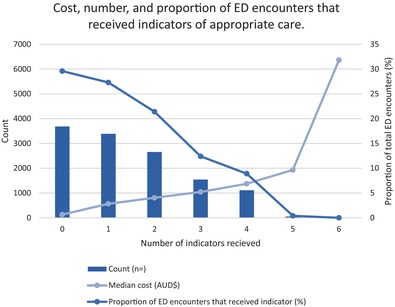

**Key findings**

- Engagement and referral to both US and psychosocial support significantly increased over the decade.
- Minority of women (9.4%, *n* = 1164/12 436), were recorded as receiving appropriate care (four or more indicators) when presenting to the ED with early pregnancy bleeding.
- The low proportion of women that received recommended care in the ED demonstrates an evidence‐practice gap and need for inquiry into barriers and facilitators to ED clinicians delivering appropriate, evidence‐based care for this patient group.

## Introduction

Early pregnancy is defined as the first 20 weeks of pregnancy.^1^ When complications occur this early, up to approximately 24% result in pregnancy loss.^1^ Vaginal bleeding is the most frequent early pregnancy complication reported, most associated with fetal demise, and causes such as ectopic pregnancy can be life‐threatening.^2^ The care women do or do not receive can have immediate and long‐term health impacts.^3^

Women with early pregnancy bleeding present to the ED expecting appropriate, evidence‐based care to determine their health status and that of their baby.^4^ The term ‘appropriate care’ refers to care that is in line with consensus‐based or evidence‐based guidelines.^5^ Considerations for appropriate care include resource use and availability, equitable access to healthcare services and patient preferences.^6^ The proportion of patients with early pregnancy bleeding who receive appropriate care has not been reported.

In general, there is increasing evidence of unwarranted variation in care received and that regarded as appropriate.^5^ For example, in the CareTrack series examining the percentage of healthcare encounters that received appropriate care, a 2017 study investigating lower back pain care, found compliance with practice guidelines ranged from 83% (care received in hospital) to 54% (care provided by general practitioners).^7^

Unwarranted clinical variation in care is a national healthcare delivery issue. In 2015, the Australian government launched the Atlas of Healthcare Variation Series.^8^ Data are derived from routinely gathered information, and differences are mapped across the country, with the aim to prompt further inquiry. The Atlas Series reviews maternity and women's health as a ‘theme’; however, there is no focus on pregnancy loss, or early pregnancy complications, which signifies a gap in evidence.^8^

Over‐, under‐ or misuse of healthcare services is a barrier to quality healthcare, and care deviations reinforce disparities that contribute to poor health outcomes.^6^ Women with early pregnancy bleeding continue to report dissatisfaction with care following presentation to ED, this could be suggestive of care not being appropriate, by not meeting their needs.^3^, ^9^ Appropriate care for women presenting to the ED with early pregnancy bleeding would include blood tests, US and referrals to services such as outpatient clinics for psychosocial support.^10^, ^11^, ^12^ The proportion of women who receive appropriate care following presentation to ED for early pregnancy bleeding is explored in the present study.

### Objective

The aim of the present study is to determine the proportion and characteristics of women presenting to EDs across a regional health district with early pregnancy bleeding, who received appropriate care.

## Methods

### Study design and setting

This is a retrospective data linkage cohort study for women of reproductive age, who presented to one of five EDs across a rural regional health district, with early pregnancy complications including vaginal bleeding, between 1 January 2011 and 31 December 2020. The present study used a clinical and system perspective of appropriate care, where outcomes were compared against practice guideline adherence across and between geographic regions and healthcare facilities, with considerations for cost effectiveness, and reduced variation in resource use.^6^

The health district is located south of Sydney, in NSW Australia. There were five EDs operating during the study period, including a level 6 tertiary, level 6, level 3 and two level 2 (one rural) EDs^13^ (Table S1). The health district has 150 000 ED presentations annually.^14^ In 2012, the level 6 tertiary site gained an Early Pregnancy Assessment Service (EPAS). The number of women presenting to the health district with early pregnancy complications, such as bleeding, increased by 19.6%, from 1322 in 2011 to 1581 in 2020.^15^

### Identification of indicators

A review of primary research and expert consensus guidelines was completed in 2023 to identify indicators of appropriate care for early pregnancy bleeding in ED. A total 117 publications (73 primary research, 38 reviews and six practice guidelines) resulted in identification of seven indicators of appropriate care grouped to three categories; assessment, intervention and diagnostic management of early pregnancy bleeding. The indicator, category, contextual considerations and corresponding evidence are listed in Table 1. The full search strategy, analysis and consolidation is published elsewhere.^16^

**TABLE 1:** Indicators of appropriate ED care of early pregnancy bleeding

Indicator of appropriate care	Consideration	Limitations
Patient received blood test: full blood count (Hb, WCC, platelet) during *ED encounter* ^10^	Only applicable to women who are haemodynamically unstable (require resuscitation). Triage category used as a surrogate indicator of haemodynamic status. Data analysis restricted to patient who received triage category 1 (life‐threatening requiring immediate care) and 2 (emergency, care within 10 min)^17^	
Patient received blood test: blood group and antibody screen during *ED encounter* ^11^, ^18^	Recommended as a baseline assessment	Antibody screening could have been performed before presentation, clinically unlikely to be repeated.
Patient received blood test: cross‐match during *ED encounter* ^10^	Only applicable to women who are haemodynamically unstable (require resuscitation). Triage category used as a surrogate indicator of haemodynamic status. Data analysis restricted to patients who received triage category 1 (life‐threatening requiring immediate care) and 2 (emergency, care within 10 min).^17^	Cross‐match could have been performed before presentation, clinically unlikely to be repeated.
Patient received blood test: βhCG during *ED encounter* ^10^, ^19^, ^20^	Recommended as a baseline assessment	βhCG could have been performed before presentation, clinically likely to be repeated.
Patient received US during *ED encounter* ^10^, ^20^	Recommended as a baseline assessment	US could have already been attended before presentation to ED, clinically likely to be repeated if resource available.
Patient received referral to or psychosocial support by health service (i.e. social worker and/or Early Pregnancy Assessment Service (EPAS) during *ED encounter* ^19^, ^21^	Always applicable (not available at all hospitals, other support services available). Data from social worker and outpatient clinics such as EPAS used.	Women need to have access to psychosocial support, does not necessarily have to be health‐service‐based. Psychosocial support could be social media groups, community support groups or private psychologist.
Patient with negative blood group received Rhesus D immunoglobulin during *ED encounter*.^11^, ^18^	Only applicable to women who have Rhesus D negative blood, and an identified synthesising event.	According to Victorian Department of Health (26), 17% of women in Australia have Rhesus D negative blood group.

### Participants

All female patients of reproductive age (10–50 years),^2^ who presented to one of the five EDs, with a recorded presenting problem of vaginal bleeding in early pregnancy, between 1 January 2011 and 31 December 2020, were included.

### Data collection, management and analysis

Five data sets were extracted from a local databank of electronic non‐identifiable patient records at the Health District. These data sets included ED, pathology, radiology, costs and non‐admitted/Outpatient. ED data set was identified as the core set, used to apply inclusion/exclusion criteria and as base for linking. Cost data were provided from the local health district costing unit and was available from 2014 to 2020 only. Overhead corporate, direct and indirect costs for each ED encounter were extracted and a sum amount calculated by Power Performance Manager (PPM2), and in accordance with Australian Hospital Patient Costing Standards (version 8.0).

**Figure 1:**
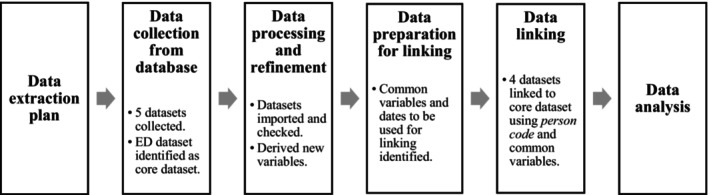
Multistep process of data collection, management and analysis.

SAS statistical software (version 9.4) was used for data processing and deterministic linking. Data were imported, checked, exclusions applied and new variables derived, for example, delays for diagnostic assessments (refer to Table S2 for data set details). The final linked data set was analysed using jamovi (version 2.3.12). The six‐step process for data collection, management and analysis is outlined in Figure 1.

Descriptive statistics were used to summarise patient characteristics. Median and interpercentile ranges were used to summarise data that were not normally distributed.^22^ Eligible encounters were calculated from the number of presentations to the ED for early pregnancy bleeding. The percentage of women that received recommended care during ED encounter was calculated as a proportion of eligible encounters. Cell size suppression was practiced when findings met the threshold (<5), to prevent backwards tracing.^22^

### Main outcomes measured

Proportion of patients that received the seven indicators of appropriate care during their ED encounter (Table 1). Secondary outcome measures included factors that affect compliance with appropriate care such as ED facility and year of presentation.

### Ethical approvals

Ethical approval was granted by the Local Health District (ISLHD/LNR/2021‐14).

## Results

### Demographics of participants

There were 9859 women who presented with early pregnancy vaginal bleeding to one of five EDs, with 12 436 eligible ED encounters, over the 10‐year study period. The mean age at time of presentation was 29.1 years (standard deviation [SD] 7.3), and the average age increased from 28.5 years (SD 7.4) in 2011 to 29.2 years (SD 7.1) in 2020. The largest proportion of women were aged between 25 and 34 years (48.8%, *n* = 7148). Most women were born in Australia (86.1%, *n* = 8466), with the remaining born across 124 countries (Table 2). The leading preferred language was English, and 1.9% (*n* = 187) of women requested a translator on arrival to ED. Of the women who presented to ED, 7.7% (*n* = 761) identified as Aboriginal and/or Torres Strait Islander.

**TABLE 2:** ED presentation and participant demographic characteristics by facility of presentation

Characteristic†	Facility of presentation				
	Level 2	Level 2 (rural)	Level 3	Level 4	Level 6
Total number ED encounters for early pregnancy bleeding, *n* (%)	50 (0.4)	1039 (8.4)	2151 (17.3)	3246 (26.1)	5950 (47.8)
Average age in years (SD)‡	30.9 (7.8)	29.4 (6.8)	28.6 (7.5)	28.5 (7.4)	29.7 (7.2)
Country of birth, *n* (%)‡					
Australia	38 (92.7)	714 (90.4)	1534 (91.5)	2486 (90.9)	3921 (81.1)
England	§	11 (1.4)	23 (1.4)	34 (1.4)	61 (1.3)
New Zealand	§	8(1.0)	24 (1.4)	28 (1.1)	66 (1.4)
China (excludes SARs and Taiwan)	§	§	§	12 (0.5)	98 (2.0)
India	§	6 (0.8)	6 (0.4)	16 (0.6)	61 (1.3)
Other (*n* = 119 countries)	§	49 (6.2)	89 (5.3)	137(5.5)	619 (12.8)
Indigenous status, *n* (%)‡					
Aboriginal and/or Torres Strait Islander origin	§	63 (0.5)	184 (1.5)	474 (3.8)	323 (2.6)
Neither Aboriginal and/or Torres Strait Islander	47 (0.4)	967 (7.8)	1960 (15.8)	2766 (22.2)	5609 (45.1)

† Excludes unknown, missing and not stated data – Indigenous status (0.6%), country of birth (0.3%).

‡ As reported at index presentation to the ED.

§ Cell size suppression threshold met; data withheld to maintain privacy (*n* < 5).

### Analysis of appropriate care

#### Number of indicators received

No women were recorded as receiving all seven indicators of appropriate care, and 54 (0.4%) received five or more indicators during their ED encounter (Table S3). During almost one third of ED encounters (*n* = 3661, 29.4%) women received no indicators of appropriate care. Women who presented to the rural facility had the lowest proportion of indicators received, with 95.5% (*n* = 992) receiving less than three indicators of care, compared to the level 6 facility where 71.0% (*n* = 4227) of women received less than three. Over the study period, the proportion of women that received an indicator of care increased, from 34.6% (*n* = 388) in 2011 to 84.4% (*n* = 1155) in 2020.

#### Summary of results

##### Psychosocial support referral or care

One third of women were referred to and/or accessed health service‐based psychosocial support, for example, social worker and midwifery run assessment units (Table 3). Of the women who received support 23.9% (*n* = 2966) received two or more interactions. Involvement of psychosocial support increased by 490.0% over the study period, with referral and/or accessed increased from 97 encounters and 8.6% in 2011, to 581 and 42.5% in 2020.

**TABLE 3:** Number and type of indicator received by facility of presentation

Indicator received during *ED encounter*	Number of eligible ED encounters (*n*)	Number of encounters excluded (*n*)†	Total number of indicators performed (*n*)	Overall compliance (%)	Facility of ED encounter compliance count, *n* (proportion [%])				
					Level 2	Level 2 (rural)	Level 3	Level 4	Level 6
Full blood count	388	12 048	228	58.8%	‡	32 (39.0)	52 (61.2)	31 (62.0)	112 (66.7)
Blood group and antibody screen	12 436	Nil	3252	26.1%	11 (22.0)	138 (13.3)	615 (28.6)	805 (24.8)	1683 (28.3)
Cross‐match	388	12 048	40	10.3%	‡	6 (7.3)	8 (9.4)	8 (16.0)	17 (10.1)
βhCG	12 436	Nil	5588	44.9%	17 (34.0)	92 (8.9)	1093 (50.8)	1446 (44.5)	2940 (49.4)
US	12 436	Nil	4641	37.3%	19 (38.0)	51 (4.9)	1124 (52.3)	452 (13.9)	2995 (50.3)
Psychosocial support	12 436	Nil	4189	33.7%	21 (42.0)	294 (28.3)	825 (38.4)	472 (14.5)	2577 (43.3)
Anti‐D injection	2114	10 322	158	7.5%	0 (0.0)	17 (9.6)	16 (4.4)	63 (11.4)	62 (6.1)

† Number of records excluded based on need for indicator: haemodynamically stable excluded (*n* = 12 048) for two indicators, and positive blood Rhesus D antigen (*n* = 10 322).

‡ Cell size suppression threshold met; data withheld to maintain privacy (*n* < 5).

##### Administration of Rhesus D immunoglobulin

Rhesus D immunoglobulin (anti‐D) was administered 158 times to women who presented to the ED. It is estimated that 17% of Australian women have a negative blood^23^ and therefore would require anti‐D if a sensitising event were to occur, such as bleeding.^18^ The number of eligible ED encounters was calculated, and 17% of the total number of ED encounters equates to 2114. The expected proportion of women receiving anti‐D would be in line with the estimated proportion of Australian population with a negative blood group. Administration of anti‐D was 7.5% and was higher in rural facilities, with level 2 and level 4 facilities having 9.6% and 11.4%, respectively (Table 3). This did not change significantly over the study period.

##### Full blood count – restricted to categories 1 and 2

Of the 388 eligible ED encounters, it was recorded that 228 (58.8%) received a full blood count pathology test. This did not change significantly over the study period.

##### Blood group and antibody screen

Overall, 26.1%, and 3252 eligible ED encounters received blood group and antibody screening. The number and rates of ordering blood group and antibody screening blood tests increased by 180.6% over the study period, from 18.7% in 2013 (*n* = 216) to 45.8% in 2019 (*n* = 606).

##### Cross‐match and hold

Of the 388 eligible ED encounters, 40 received a cross‐match and hold pathology testing, representing 10.3% proportion of women. This did not change over the study period.

##### βHCG

Only 44.9% of women received a βhCG blood test (Table 3). βhCG testing increased by 170.6% over the study period, from 27.6% (*n* = 310) in 2011 to 61.3% (*n* = 839) in 2020.

##### Ultrasound

Only 37.3% (*n* = 4641) of women received an US during their ED encounter (Table 3). Of those women, 27.3% (*n* = 3389) received a single US, and 10.1% (*n* = 1252) received two or more. The number of USs increased by 233.3% over the study period, from 225 in 2012, to 750 in 2020. Overall proportion of women receiving US increased from 18.6% in 2012 to 54.8% in 2020.

##### Costs

**Figure 2:**
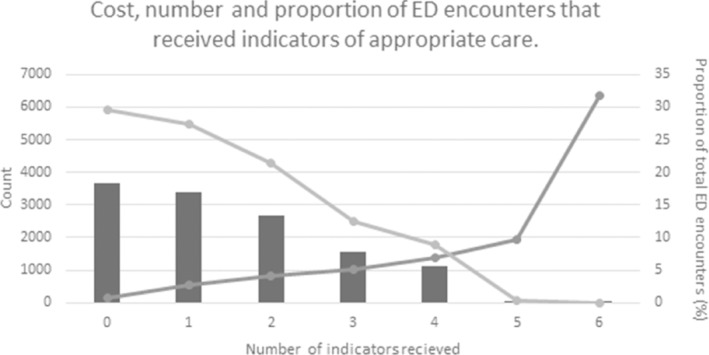
Cost, number and proportion of ED encounters with early pregnancy bleeding that received indicators of appropriate care. (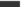), Count (*n*); (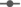), median cost (AU$); (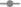), compliance (%).

Median cost of each ED encounter increased with proportion of women receiving recommended care (Fig. 2), from AU$566.00 (interquartile range [IQR] AU$1089.50) when one indicator was received, to AU$1930.00 (IQR AU$3286.75) median cost when patients received five or more indicators (Table S3).

## Discussion

One third of women who presented to ED with early pregnancy bleeding did not receive any indicators of appropriate care. The indicator that was received by the highest recorded proportion of women was full blood count (58.8%, *n* = 228) and the lowest was anti‐D injection administration (7.4%, *n* = 157). Both US and psychosocial support referrals and engagement significantly increased over the study period. This may be explained by the opening of an EPAS in 2012, attached to one study site, that accepted referrals from across the health district.^16^ Evidence suggests EPASs have a positive impact on health service outcomes including reduced ED presentations, length of stay and cost and women's experiences.^1^, ^3^ Meeting patients' expectations, not only boosts their experience and satisfaction, but can also reduce the negative impact of early pregnancy bleeding which can be a distressing life event.^4^

### Reasons for deviation from appropriate care

Less than 10% of women received four or more indicators, despite at least four indicators being recommended for all 12 436 ED encounters.^16^ This could be explained by variations in patient preferences and values, resource availability, guideline and data limitations.

#### Patient perspective

A person‐centred approach to care respects and responds to the preferences, needs and values of patients, and has been widely accepted as fundamental to safe and high‐quality healthcare.^8^ A pregnancy becomes complex when women experience biomedical or psychosocial risk factors that put mother and/or baby at increased risk of adverse outcomes such as vaginal bleeding.^24^ Women with a complex pregnancy have less access to care that is person‐centred and are at higher risk for poor outcomes or ‘falling through the gaps’ in the healthcare system.^24^ Reasons for this disparity include lack of guidelines or polices resulting in fragmented care with unclear pathways for referral and follow up, and lack of number, capacity and linked specialist services, when multispecialist input and crossing multidisciplinary boundaries is needed.^24^

#### Variations in resource availability

Almost 90% of women who presented to the level 2 rural facility received less than one indicator of care during their ED encounter, compared to 50% of women in level 6 referral facility. Challenges to reduced access and receipt of healthcare in rural and remote Australia have been widely documented.^25^ Inequities in resource availability for rural communities such as limited access to specialist care, for example, EPAS, or differing models of care, for example, restricted medical or pathology staffing hours, is an obvious barrier to appropriate care.^3^ However, in the present study, it is not known if interventions and/or diagnostics were performed at private facilities or if women were transferred to another hospital (in or outside the health district) for preliminary management, and this is a limitation to retrospective data use.

#### Guideline limitations and gaps

Clinical practice guidelines (CPGs) are intended to optimise patient care and present systematically developed recommendations for clinician and patient decisions.^26^ A lack of CPGs, out of date CPGs or vague recommendations can cause clinicians to misinterpret and overlook vital aspects of appropriate care.^6^ For example, nonspecific recommendations for appropriate analgesia for women experiencing pain with bleeding, can contribute to lower rates of analgesic administration.

The study site did not have a guideline for assessment, intervention and diagnostics for early pregnancy bleeding in the ED. Further, a 2023 review of ED care of early pregnancy bleeding found that 12% of CPGs and practice statements include outdated recommendations.^16^ The quality of CPGs should be assessed for clinical credibility and trustworthiness, and the potential use of implementation tools such as AGREE‐REX could be considered.^27^

Implementation of CPGs often requires significant changes in clinician behaviour, with the introduction of new technologies and alterations to the ‘usual’ care provided.^28^ There are many recognised barriers to behaviour change, including unsupportive organisational culture, clinicians' attitudes and beliefs, and competing interests.^28^ These challenges can contribute to deviations from appropriate care.^5^ Application of behaviour‐change processes to the implementation of CPGs informed by theory is emerging.^28^ Further research is needed to understand aspects of clinicians' perspectives on clinical practice and appropriate care, and how CPGs can be implemented more effectively.

#### Impact of SARS‐CoV‐2 pandemic

Study period included approximately 9 months of SARS‐CoV‐2 pandemic, during this time the global healthcare systems were adapting and responding to new demands and evolving strategies of working.^29^ ED presentations declined over Australia and internationally,^30^ and could explain some variations in indicators received over the study period. Reasons included fear of exposure to SARS‐CoV‐2 in healthcare facilities, and access to at‐home or virtual care.^30^

### Use of data

Digitalisation of health records has contributed to generation of large data registries.^31^ Data linkage is a strategy to combine digital information across platforms to map a patient's pathway throughout their healthcare interaction and be cost effective, non‐intrusive and overcome limitations such as data splitting due to different service types and jurisdictions.^31^, ^32^ Use of data for monitoring appropriate care should be a fundamental practice.^5^ Insights gained can inform quality and safety improvement strategies and determine unwarranted variation in care to aid in identifying evidence‐practice gaps. However, it is not without its limitations as exhibited in the present study.

### Limitations

Retrospective data relies on accurate documentation and is inherently biased. For example, data were unavailable for care that women received before presenting to the ED. With the expansion of community‐based clinics and private US facilities, women often present to the ED having already had an US and with report in hand. With continued investment and expansion of electronic medical records and data linking strategies, there is growing potential in the use of patient record data for care audits. Results may not be generalisable to other geographical areas as the present study is illustrative of the population within one health district in Australia. The nature of assessments performed, and variety of methods for documentation for this unique population, limited availability of data, and it was not feasible to perform a hand search and review of all 12 436 records. Furthermore, the patient perspective and considerations of patient preferences and values were largely neglected, as there was no formal documentation of such information.

## Conclusions

Using linked data, variation and often absence ED care for women presenting with early pregnancy bleeding were identified, where only 10% of women received four or more indicators of appropriate care and one in three women received zero. Demonstrating an evidence‐practice gap and need for inquiry into barriers to prescribed clinical practice for this vulnerable and unique population.

## Supporting information


**Table S1.** NSW role delineation levels of emergency departments adapted from the role delineation of clinical services by the Ministry of Health, 2021.
**Table S2**. Extracted data set name, variables extracted and linking terms used to generate linked data set.
**Table S3**. Cost, number of indicators received and compliance by faculty of ED encounter.

## Data Availability

The data that support the findings of the present study are available from Centre for Health Research Illawarra Shoalhaven Population. Restrictions apply to the availability of these data, which were used under licence for the present study. Data are available from corresponding author with the permission of Centre for Health Research Illawarra Shoalhaven Population.
