# Supplementary material for: What proportion of women presenting to the emergency department with early pregnancy bleeding receive appropriate care?
Source: Emerg Med Australas. 2024 Oct 7;37(1):e14507. doi: 10.1111/1742-6723.14507 (PMC11744408; doi:10.1111/1742-6723.14507)
Supplement: Supplementary file 1 — Table S1. NSW role delineation levels of emergency departments adapted from the role delineation of clinical services by the Ministry of Health, 2021. Table S2. Extracted data set name, variables extracted and linking terms used to generate linked data set. Table S3. Cost, number of indicators received and compliance by faculty of ED encounter. [file EMM-37-0-s001.docx]

## Table S1: NSW role delineation levels of emergency departments adapted from the role delineation of clinical services by the Ministry of Health, 2021 ^13^.

| **Emergency Department delineation** | **Level description and services available** |
| --- | --- |
| Level two | These EDs provide limited radiology including x-ray, and pathology services. Perform stabilisation of critically ill paediatric, adult and trauma patients prior to retrieval service, including basic primary and secondary assessment. |
| Level three | All services listed in “level 2”, plus: provide radiology including ultrasound, and pathology services. These EDs manage complex case and provide emergency care for example short term ventilation (waiting for transfer). |
| Level four | All services listed in “level 3”, plus: radiology including 24 hours access to ultrasound, and pathology services. These EDs offer definitive care for most presentations. |
| Level six | These EDs radiology including 24 hours access to ultrasound, and large range of tests available for pathology services. They are a referral centre to lower-level EDs and can manage all complex cases. |

## Table S2: Extracted dataset name, variables extracted and linking terms used to generate linked dataset.

| **Name of dataset** | **Variables extracted** | **Variables used for linking to core dataset (ED)** |
| --- | --- | --- |
| Emergency Department | Person code, visit code, facility of presentation (identifier code), actual departure date and time, age, arrival date and time, country of birth, SNOMED diagnostic code, type and description, ED source of referral, first date and time seen by clinician and nurse, Indigenous status, mode of arrival and separation, need for interpreter presenting problem, referred to on departure, sex, triage category, triage date and time, | Person code, arrival, and actual departure date. |
| Pathology | Person code, facility of presentation (identifier code), episode code, request date, collection time, service date and time, location code, item described. | Person code, request date. |
| Radiology | Person code, facility of presentation (identifier code), episode code, procedure performed description, date and time seen. | Person code, date seen. |
| Costs | Person code, facility of presentation, start and discharge date, category of cost (e.g., allied, medical, nurse), sum of costs. | Person code, start discharge date. |
| Non-Admitted/ Outpatient | Person code, service event code, service/ unit type code and full name, service start and end date and time, service contact mode. | Person code, service start date. |

## Table S3: Cost, number of indicators received and compliance by faculty of ED encounter.

| **Number of indicators received during ED encounter.** | **Raw compliance count (n=) (proportion (%))** | **Cumulative compliance count (n=) (proportion (%))** | **Facility of ED encounter compliance count (n=) (proportion (%))** | | | | | **Median cost (IQR) (AUD$)** |
| --- | --- | --- | --- | --- | --- | --- | --- | --- |
|  |  |  | **Level two** | **Level two (rural)** | **Level three** | **Level four** | **Level six** |  |
| **7** | 0 (0) | 0 (0) | 0 (0) | 0 (0) | 0 (0) | 0 (0) | 0 (0) | 0 (0) |
| **6** | ** | ** | 0 (0) | ** | 0 (0) | 0 (0) | ** | 6361 (3180.50–9541.50) |
| **5** | 52 (0.4) | 54 (0.4) | 0 (0) | 0 (0) | 8 (0.4) | ** | 40 (0.7) | 1930 (1130-4382.25) |
| **4** | 1110 (8.9) | 1164 (9.4) | 7 (14.0) | 8 (0.8) | 248 (11.5) | 40 (1.2) | 807 (13.6) | 1374 (889-2608.50) |
| **3** | 1544 (12.4) | 2708 (21.8) | ** | 38 (3.7) | 346 (16.1) | 282 (8.7) | 875 (14.7) | 1042 (629-1972.50) |
| **2** | 2687 (21.6) | 5395 (43.4) | 9 (18.0) | 89 (8.6) | 548 (25.5) | 633 (19.5) | 1408 (23.7) | 802 (0-  1641) |
| **1** | 3380 (27.2) | 8775 (70.6) | 15 (30.0) | 300 (28.9) | 568 (26.4) | 986 (30.4) | 1511 (25.4) | 566 (0-1089.50) |
| **0** | 3661 (29.4) | 12436 (100.0) | 16 (32.0) | 603 (58.0) | 433 (20.1) | 1301 (40.1) | 1308 (22.0) | 124 (0-584) |
| **Total number of ED encounters** | 12436 |  | 50 | 1039 | 2151 | 3246 | 5650 | 626.50 (0-1315.25) |
| **Sum of eligible ED encounters** | 52634 |  | 215 | 4497 | 9140 | 13636 | 25148 |  |
| **Sum of all ED encounters** | 87052 |  | 350 | 7273 | 15057 | 22722 | 41650 |  |
| **Sum of excluded records* (proportion of all ED encounters (%))** | 34418 (39.5) |  | 135 (38.6) | 2776 (38.2) | 5917 (39.3) | 9086 (40.0) | 16502 (39.6) |  |
| *Number of records excluded based on need for indicator: haemodynamically stable excluded (n=12048) for two indicators, and positive blood Rhesus D antigen (n=10322). **Cell size suppression threshold met; data withheld to maintain privacy (n<5). | | | | | | | | |
